# Filariasis: a vasculitis mimic

**DOI:** 10.1093/rap/rkz045

**Published:** 2019-12-17

**Authors:** Rajiv Ranjan Kumar, Anu Balakrishnan, Shiv Kumar Suman, Sauvik Dasgupta, Nitin Gupta, Archana G Vallonthaiel, Sudheer Arava, Bijay Ranjan Mirdha, Uma Kumar

**Affiliations:** 1 Department of Rheumatology, New Delhi, India; 2 Department of Medicine and Microbiology, New Delhi, India; 3 Department of Pathology, New Delhi, India; 4 Department of Microbiology, AIIMS, New Delhi, India

**Key message**

- Infective aetiologies must be retained in differential diagnosis of acute onset arthritis and gangrene.

Sir, Filariasis is an important cause of morbidity in highly endemic areas, which span throughout the tropical and sub-tropical countries of Asia, Africa and the western Pacific. The World Health Organization estimates that nearly 1.4 billion people in 73 countries worldwide are threatened by filariasis. Lymphatic filariasis is commonly caused by *Wuchereria* *brancrofti*, followed b*y Brugia malayi* and *Brugia timori* [1]. The disease has a number of varied presentations, ranging from asymptomatic infection to acute dermatolymphangitis or chronic features of lymphatic obstruction [2]. We report this case to highlight the fact that common diseases may present with very unusual manifestations in an endemic setting.

**Fig. 1.**
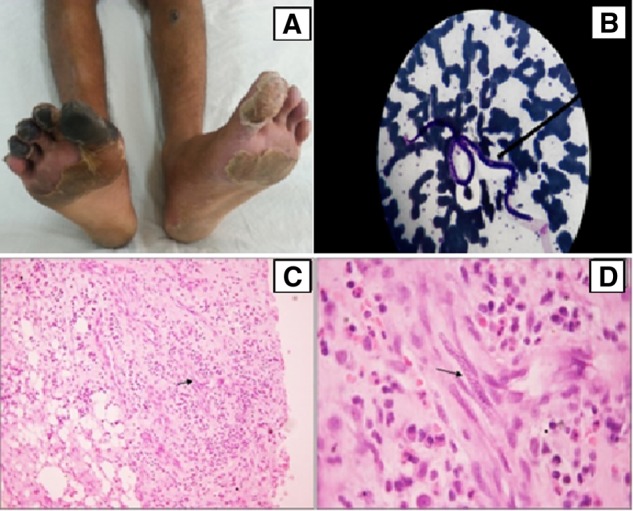
Filariasis: a vasculitis mimic (A) Ulcerated nodule over left shin and dry gangrene of great toe of right leg, with gangrene of tip of other toes of right leg and excoriation of skin of both legs. (**B**) The Giemsa staining of blood, showing microfilaria of size ∼240–250 µm in length and 7–9 µm in width. (**C**) Skin biopsy (from nodule) showing dense inflammation involving dermis and subcutaneous fat. Capillary proliferation is noted, one of which shows the presence of microfilaria (arrow) (×100; Haematoxylin and Eosin). (**D**) Capillary lumen, showing presence of microfilaria (arrow) surrounded by inflammatory cells including plasma cells, lymphocytes and neutrophils (×1000; Haematoxylin and Eosin ).

We report the case of a 19-year-old male, resident of Jharkhand (a state in eastern India), who presented with acute onset right knee joint arthritis along with dry gangrene of the right great toe for 1 month without any history of fever, weight loss, oral ulceration, RP, alopecia, skin rash or sicca symptoms. There were no other systemic features. Examination revealed mild pallor, bilateral painless inguinal lymphadenopathy (1 cm×1 cm), bilateral non-tender nodules over the shin (0.5 cm × 0.7 cm; one of which was ulcerated), right knee arthritis, dry gangrene of the right great toe (up to the level of the metatarsophalangeal joint) and gangrene of the tips of the other toes of the same side (Fig. 1A).

Laboratory examination showed mild anaemia (microcytic, hypochromic), eosinophilia (absolute eosinophil count 1545/mm^3^), platelet count of 4.5 lakhs/mm^3^, erythrocyte sedimentation rate of 55 mm/h and CRP of 15.2 mg/l. Renal, liver and cardiac function were normal. Autoimmune markers were also non-contributory (ANA-1: 40, speckled, RF/Anti CCP-negative, normal complement, negative for anti-dsDNA, ANCA and antiphospholipid antibodies). Knee joint synovial fluid revealed neutrophilic leucocytosis (cell count 12 000/mm^3^, with 90% polymorphs), sterile on culture and negative for crystals. Synovial fluid Gram stain and Ziehl–Neelsen stain were negative. Doppler US of bilateral lower limbs revealed dampened and monophasic flow bilaterally in the anterior tibial arteries, suggestive of vasculitis involving the distal arteries. CT of the abdomen and angiography of the abdominal aorta and its branches were unremarkable. Quantitative buffy coat examination of blood revealed microfilariae (Fig. 1B), which were identified as those of *W. brancrofti* based on their micromorphological features. A skin biopsy from the ulcer edge showed dense inflammation involving the dermis and subcutaneous fat, capillary proliferation and the presence of microfilariae (Fig. 1C and D).

A diagnosis of filariasis was made, and the patient was treated with diethylcarbamazine 100 mg three times daily for a total of 3 weeks. The patient experienced complete recovery from the arthritis and gangrene, except for the gangrene of the great toe, which required cosmetic amputation.

To the best of our knowledge, this is the first case report of filariasis with co-existing arthritis and gangrene. There are very few reports of musculoskeletal involvement in cases of filariasis [3–5]. A case series of 45 patients of filariasis presenting with acute arthritis was reported from Bangladesh [3]. Arthritis attributable to filariasis can manifest either as oligoarthritis or as polyarticular pseudorheumatism [6]. The knee is the most commonly affected joint in cases of filarial arthritis. The pathogenesis is believed to be either immune complex mediated or as a result of tissue reaction to a filarial worm in the vicinity of the joint [3]. The presence of microfilariae in the biopsy from the gangrenous area suggests that the microfilariae might have been responsible for the ischaemic damage to the skin. This is further evidenced by the resolution of the gangrene after the anti-filarial treatment. Although gangrene attributable to microfilariae of *W. brancrofti* is extremely rare, other vascular manifestations are shown to be associated with microfilariae. These include vasculitis, thrombosis, dilated lymphatics, lymphangitis and nodal fibrosis [7–9]. A few cases of retinal vasculitis and a case of leucocytoclastic vasculitis have also been reported [8]. Other skin manifestations that are reported in cases of filariasis include skin rashes, abscesses, nodules, granulomata and cysts [8, 9]. In developing countries with high endemicity of filariasis, atypical manifestations of this disease should be borne in mind.
